# The inconsistent mediating effect of catechol *O* methyl transferase Val^158^Met polymorphism on the sex difference of cognitive impairment in schizophrenia patients

**DOI:** 10.3389/fpsyt.2022.993859

**Published:** 2022-09-20

**Authors:** Hang Xu, Yongjie Zhou, Meihong Xiu, Dachun Chen, Weiwen Wang, Li Wang, Xiangyang Zhang

**Affiliations:** ^1^CAS Key Laboratory of Mental Health, Institute of Psychology, Beijing, China; ^2^Department of Psychiatric Rehabilitation, Shenzhen Kangning Hospital, Shenzhen, Guangdong, China; ^3^Beijing HuiLongGuan Hospital, Peking University, Beijing, China; ^4^Department of Psychology, University of Chinese Academy of Sciences, Beijing, China

**Keywords:** schizophrenia, catechol-*O*-methyl transferase (COMT), Val158Met (rs4680), sex difference, cognitive function

## Abstract

**Objective:**

Schizophrenia is a multifaceted mental disorder characterized by heterogeneous positive/negative symptoms and cognitive deficits. Sex differences have been reported in various aspects of the disease. However, the underlying genetic reasons remain unelucidated. Recent studies show that the influence of *COMT* Val158Met (rs4680) variation is sexually dimorphic. Thus, this study aims to explore whether there is an effect of the interaction between *COMT* Val158Met (rs4680) polymorphism and sex on patients’ clinical characteristics and cognitive function.

**Materials and methods:**

We recruited 367 in patients with chronic schizophrenia (246 males and 121 females) and 419 healthy controls (172 males and 247 females). The cognitive performance was assessed by Repeatable Battery for the Assessment of Neuropsychological Status (RBANS), and the *COMT* Val158Met (rs4680) polymorphism is genotyped. The psychopathological symptoms of the patients were assessed by the Positive and Negative Syndrome Scale (PANSS).

**Results:**

We find that male patients had a significantly higher proportion of carrying the Val allele and Val/Val carriers exhibited more severe positive symptoms and cognitive impairment than Met carriers. *COMT* Val158Met (rs4680) polymorphism inconsistently mediated the relationship between sex and cognitive performance in schizophrenia patients.

**Conclusion:**

These findings suggest that *COMT* Val158Met (rs4680) polymorphism is associated with the risk and severity of schizophrenia in a sexually dimorphic way and contributes more to the clinical symptoms and cognitive impairment in male patients with schizophrenia.

## Introduction

Schizophrenia is a devastating mental disorder, characterized by heterogeneous positive and negative symptoms (such as hallucinations, delusions, and reduced expression of emotions or motivation), and cognitive dysfunction (such as difficulties with concentration, working memory, and decision making) (1). Clinical evidence shows that men and women are different in prevalence, age of onset, symptomatology, treatment outcome, and neurological abnormalities (2–4). Male patients show an earlier age at onset, and more negative symptoms, while female patients display more affective, paranoid, and periodic psychiatric symptoms (4). Cognitive deficits are associated with poor functional outcomes and unfavorable prognosis. Studies show male schizophrenic patients have more serious cognitive deficits than females on multiple cognitive tasks, such as working memory, problem-solving, and verbal and visual learning (5, 6). However, the underlying molecular mechanism of sex differences for the heterogeneous features of schizophrenia remains unclear.

Catechol-*O*-methyl transferase (*COMT*) encodes a major catabolic enzyme involved in dopamine (DA) metabolism and has been widely studied in patients with schizophrenia. The human *COMT* gene is located at position 11.21 on chromosome twenty-two, which is a region closely related to mental illness. Single nucleotide polymorphism (SNP) rs4680 is one of the widely studied polymorphisms of the *COMT* gene, which transforms from valine (or allele G) to methionine (or allele A) (Val^158^Met). The available evidence has supported contradictory conclusions between *COMT* Val158Met (rs4680) polymorphism and schizophrenia. For example, Sun et al. found no significant genotypic association between Val^158^Met polymorphism and clinical symptoms or cognitive function (7), while Li et al. found that Val158Met (rs4680) polymorphism may be associated with negative symptoms of schizophrenia in Han Chinese (8, 9). One possible reason for this inconsistency is that many of these studies do not consider the sex-gene interaction.

Recent studies have reported the sexually dimorphic effect of Val158Met (rs4680) polymorphism on brain morphology. The decrease in COMT enzyme activity increases the thickness of prefrontal cortex (PFC) in male, but not female mice and humans (10, 11). Consistent with neuroanatomical changes, *COMT* Val158Met (rs4680) polymorphism is associated with sex differences in PFC-dependent working memory in patients, which means that men with the Met allele show better working memory (11). It has also been reported that there is an effect of high-activity allele loading on negative symptoms, disorganization, and cognition (such as executive function and verbal IQ) in males (12, 13), while the Met allele is associated with lower stress effects in females (14). However, the interaction of *COMT* Val158Met (rs4680) and sex on the clinical characteristics and cognitive performance in patients with schizophrenia is unclear. Based on the prior clinical, cognitive, and neuroimaging studies, we hypothesize that the *COMT* Val158Met (rs4680) variant is a potential genetic mechanism underlying the features of sex differences in schizophrenia.

Thus, this study aims to explore the effect of sex × gene interaction on the characteristics and cognitive function of patients with schizophrenia, and the mediation model is used to test whether genetic factor accounts for the relationship between sex and cognitive function in schizophrenia patients.

## Materials and methods

### Subjects

We recruited 367 inpatients (246 males and 121 females) from the Beijing Huilongguan Hospital, a Beijing city-owned psychiatric hospital. These patients were all Han Chinese and met the criteria for schizophrenia based on the Diagnostic and Statistical Manual of Mental Disorders, Fifth Edition (DSM-V). The diagnosis was independently confirmed by two experienced psychiatrists. The patients met the following criteria: (a) aged between 20 and 75 years old, with more than 5°years of education; (b) had a course of disease for at least 5 years; (c) received a stable dose of oral antipsychotic drugs for more than 1°year. Antipsychotic treatment was monotherapy, including clozapine (47.5%), risperidone (25.0%), chlorpromazine (4.9%), sulpiride (8.2%), perphenazine (6.6%), haloperidol (4.5%), nimesulide (2.9%), and quetiapine (0.4%). We excluded patients with major physical diseases or any acute or chronic diseases affecting the immune, endocrine, metabolic, or nervous systems (such as cerebrovascular disease, cancer, lung disease, or diabetes), as well as pregnant or breastfeeding women. 181 male and sixty-three female of those patients completed the cognition assessment.

In the same period, a total of 419 healthy Han Chinese were recruited from the local area in Beijing, including 172 males and 247 females. Any healthy subjects with a history of medical abnormalities or common mood disorders or substance abuse/dependence were excluded. The age, education level, marriage of patients, and healthy subjects matched. None of them suffered from substance/alcohol dependence/abuse except for smoking. 168 males and 229 females completed the cognitive assessment.

### Clinical assessment

Four psychiatrists simultaneously participated in a training course to evaluate the clinical symptoms using the Positive and Negative Syndrome Scale (PANSS). After training, the inter-observer correlation coefficient of the PANSS total score was maintained above 0.8 to ensure the reliability and consistency of the evaluation.

The cognitive function of all subjects was assessed by the Repeatable Battery for the Assessment of Neuropsychological Status (RBANS, Form A) (15). The clinical validity and test-retest reliability were confirmed in the Chinese general population and patients with schizophrenia (16). The RBANS provides a total score and five subscores of cognitive function, including immediate memory, visuospatial/constructional, language, attention, and delayed memory. The Chinese version of RBANS showed good validity (Cronbach’s α coefficient of the total scale, immediate memory, visuospatial, language, attention, and delayed memory were 0.9, 0.86, 0.68, 0.67, 0.85, and 0.80, respectively) and good test-retest reliability in China (16).

### Blood sampling and genotyping

After an overnight fast, venous blood was collected from the forearm vein of the subjects using the anticoagulant ethylene diamine tetraacetic acid (EDTA) tubes between 7:00 and 9:00 am. Genomic DNA was extracted from whole blood samples. Following the standard protocol, the *COMT* Val158Met (rs4680) polymorphism was identified by using Matrix-Assisted Laser Desorption/Ionization Time of Flight Mass Spectrometry (MALDI-TOF MS) (Sequenom Inc., San Diego, CA, USA). The amplification primers were: sense: 5’-TCACCATCGAGATCAACCCC-3’, antisense: 5’-GAACGTGGTTGTAACACCTG-3’. In addition, 5% of the samples were genotyped for error checking, with reproducibility of >0.99. *COMT* Val158Met (rs4680) polymorphism genotyping was conducted by a technician who was blind to the clinical status of the subjects (17).

### Statistical analysis

Statistical analyses were conducted using the Statistical Package of Social Sciences version 24 (SPSS, Inc., Chicago, IL, USA) for Windows. Differences between groups were explored using ANOVA for continuous variables and the chi-square test for categorical variables. The ANOVA was first conducted for omnibus effects of the COMT × sex interactions on cognition and symptoms. The differences were further confirmed by MANCOVA (controlling for different covariates). We presented the results of MANCOVA in the tables. The interaction analysis of diagnosis, sex, and COMT Val158Met (rs4680) polymorphism on cognitive functions adopted the three-way ANOVA/MANCOVA (age and education as covariates). The interaction effects of sex and genotype on cognitive functions in healthy control and patients were analyzed by two-way ANOVA/MANCOVA (age and education as covariates). The one-way MANCOVA was adopted to analyze the differences between genotypes of the same gender. The age, age of onset, and education were defined as covariates in the patients. Age and education were defined as covariates in healthy control. Hardy–Weinberg equilibrium was performed to examine genotype deviation. The Hayes’ PROCESS program (18) was used to test the hypothesis that *COMT* Val158Met (rs4680) mediated the relationship between sex and cognitive performance in schizophrenia patients. The FDR correction was performed for multiple tests and *post hoc* analysis. The Cohen’s f method was used to calculate the standardized effect size. *A priori* sample sizes were estimated based on a two-sided *F* test using the G power 3.1.9.2 program. There was at least 80% power to detect a medium effect size (0.25) with a significance level of 0.05. Therefore, the required total sample size was 158 (20 for each group) (19). Descriptive summary statistics were expressed as mean ± standard deviation (SD), and differences with *p*°<°0.05 were considered to be significant.

## Results

### Demographic characteristics and genotypic data

The demographic characteristics of the subjects are shown in Table 1. In the patient group, their average age was 50.16 ± 9.80 years (ranging from 19 to 73 years), and their average course of the disease was 27.66 ± 7.79 years (ranging from 14 to 55 years). The average education level was 9.71 ± 2.52 years. In the healthy control group, their average age was 46.21 ± 13.18 years (ranging from 16 to 70 years). The average education level was 9.2 ± 3.32 years. One-way ANOVA indicated that there was a significant age difference [*F*_(1,781)_ = 32.83, *p* < 0.01], but not in education between patients and healthy controls. In addition, there was a significant sex difference in age in the healthy control group [*F*_(1,417)_ = 6.59, *p* < 0.05], and significant sex difference in educations in both healthy control and patients [*F*_(1,413)_ = 4.17, *p* < 0.05; *F*_(1,365)_ = 5.35, *p* < 0.05]. There was no significant difference in the age of onset between male patients and female patients.

**TABLE 1:** Sample characteristics of healthy controls and chronic schizophrenia patients.

	Healthy controls			Schizophrenia patients			**^c^**Statistic, *p*
	Male (*n* = 172)	Female (*n* = 247)	**^a^**Statistic, *p*	Male (*n* = 246)	Female (*n* = 121)	**^b^**Statistic, *p*	
**Age** (year)	44.24 ± 14.87	47.58 ± 11.70	6.59, 0.01*	50.13 ± 9.30	50.22 ± 10.76	0.01, 0.93	32.83, 0.00**
**Educations** (year)	9.60 ± 3.18	8.92 ± 3.40	4.17, 0.04*	9.50 ± 2.38	10.84 ± 2.77	5.35, 0.02*	1.76, 0.18
**Age of onset** (year)				23.98 ± 6.11	25.32 ± 6.51	1.41, 0.24	
***COMT*** **allele frequency** (%)			0.03, 0.88			4.16, 0.04*	0.52,0.47
Met	95(28.1%)	140(28.9%)		158(32.1%)	60(24.8%)		
Val	247(71.9%)	354(71.7%)		334(67.9%)	182(75.2%)		
***COMT*** **genotype distribution** [(n (%)]			0.82, 0.66			10.3, 0.00**	2.27,0.32
Met/Met	12(6.2%)	14(5.1%)		21(6.1%)	12(6.8%)		
Met/Val	71(36.8%)	112(40.9%)		116(33.5%)	36(20.5%)		
Val/Val	89(46.1%)	121(44.2%)		109(31.5%)	73(41.5%)		

*^a^*Statistic: sex effect in the healthy controls.

*^b^*Statistic: sex effect in the schizophrenia patients.

*^c^*Statistic: main effect of diagnostic group.

*p*: *<0.05,^**^ <0.01.

The *COMT* Val158Met (rs4680) genotype distribution was consistent with Hardy–Weinberg equilibrium in healthy controls (χ^2^ = 2.83, df = 1, *p* = 0.24) and patients (χ^2^ = 0.03, df = 1, *p* = 0.98). The distribution of *COMT* Val158Met (rs4680) genotype and allele is summarized in Table 1. There was no significant difference in *COMT* Val158Met (rs4680) genotype (χ^2^ = 2.27, df = 2, *p*°>°0.05) and allele distribution (χ^2^ = 0.52, df = 1, *p*°>°0.05) between the healthy controls and patients. However, there were significant sex differences in the *COMT* Val158Met (rs4680) genotype (χ^2^ = 10.3, df = 2, *p* < 0.01) and allele frequency in patients (χ^2^ = 4.16, df = 1, *p* < 0.05), showing that male patients had a higher proportion of Met alleles. However, there was no such sex difference in healthy controls (χ^2^ = 0.82, df = 2, *p*°>°0.05; χ^2^ = 0.03, df = 1, *p*°>°0.05, respectively). The comparisons of the genotypes/alleles between cases and controls of the same sex were performed. Significant differences in the distribution of genotypes in males between cases and controls were observed (χ^2^ = 89.93, df = 1, *p* < 0.001). And significant differences in the distribution of alleles in females between the two groups were observed (χ^2^ = 8.97, df = 1, *p* < 0.05).

### Interaction of diagnosis, sex, and catechol *O* methyl transferase Val158Met (rs4680) genotype on the cognitive function in controls and patients

Table 2 shows the results of the three-way MANCOVA interaction analysis results of the diagnosis, sex, and genotype. The main effects of diagnosis were significant in all cognitive indexes (all *p* or FDR corrected *p* < 0.05; Cohen’s *f* = 0.54, 0.23, 0.49, 0.30, 0.70, and 0.47 respectively in immediate memory, visuospatial/constructional, language, attention, delayed memory, and total score). This sample gave a statistical power of > 0.9. There was significant sex effect in delayed memory [*F*_(1,631)_ = 5.18, *p* < 0.05, FDR corrected *p* = 0.02, Cohen’s *f* = 0.18]. There was significant COMT genotype effect on language [*F*_(1,631)_ = 4.92, *p* < 0.05, FDR corrected *p* = 0.03, Cohen’s *f* = 0.18]. There was significant diagnosis × sex interaction in delayed memory [*F*_(1,631)_ = 5.58, *p* < 0.05, FDR corrected *p* = 0.09, Cohen’s *f* = 0.19], and diagnosis × COMT interaction in language [*F*_(1,631)_ = 4.77, *p* < 0.05, FDR corrected *p* = 0.10, Cohen’s *f* = 0.17], and sex × COMT interaction [*F*_(1,631)_ = 4.54, *p* < 0.05, FDR corrected *p* = 0.09, Cohen’s *f* = 0.19] in immediate memory [*F*_(1,631)_ = 4.54, *p* < 0.05, FDR corrected *p* = 0.11, Cohen’s *f* = 0.17], but those differences did not pass the correction.

**TABLE 2:** Interaction of diagnosis, sex, and catechol *O* methyl transferase Val158Met (rs4680) genotype on the cognitive performance.

Variables	Immediate memory		Visuospatial/constructional		Language		Attention		Delayed memory		RBANS total score	
	*F*	*p*	*F*	*p*	*F*	*p*	*F*	*p*	*F*	*p*	*F*	*p*
Diagnosis (D)	46.66	0.00**	7.99	0.00**	38.18	0.00**	14.61	0.00**	76.70	0.00**	34.52	0.00**
SEX	3.37	0.07	1.33	0.25	0.03	0.85	0.01	0.94	5.18	0.02*	2.54	0.11
COMT	1.15	0.28	0.02	0.90	4.92	0.03*	0.44	0.51	0.17	0.68	0.21	0.65
D × SEX	0.34	0.56	0.31	0.58	0.18	0.67	0.00	1.00	5.58	0.02*	1.44	0.23
D × COMT	1.85	0.17	0.56	0.45	4.77	0.03*	0.11	0.74	1.05	0.31	1.43	0.23
SEX × COMT	4.54	0.03*	2.97	0.09	0.53	0.46	2.68	0.10	2.62	0.11	2.40	0.12
D × SEX × BDNF	1.28	0.26	1.36	0.24	0.26	0.61	1.15	0.28	1.81	0.18	1.02	0.31

Three-way MANCOVA with age and education as the covariates; *p*: * < 0.05, ** < 0.01.

### Interaction of sex and catechol *O* methyl transferase Val158Met (rs4680) genotype on the clinical characteristics of schizophrenia patients

**FIGURE 1:**
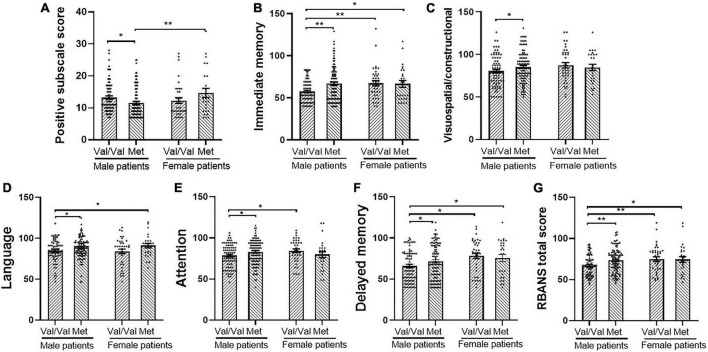
Comparisons between catechol *O* methyl transferase Val158Met (rs4680) genotype and sex with *post hoc* test on positive symptom and cognitive performance. **(A)** Positive and negative syndrome scale (PANSS) positive subscale score, **(B)** immediate memory, **(C)** Visuospatial/constructional, **(D)** Language, **(E)** Attention, **(F)** Delayed memory, and **(G)** Repeatable battery for the assessment of neuropsychological status (RBANS) total score. Error bars: standard error of the mean. *p*: * < 0.05, ^**^ < 0.01. Met means Met carrier.

The results of the analysis of the interaction effect of sex and *COMT* Val158Met (rs4680) polymorphism on the clinical characteristics of patients with schizophrenia are summarized in Table 3. After adjusting for age and education, two-way MANCOVA showed that there was a significant sex × genotype interaction effect on the positive subscale score [*F*_(1,238)_ = 7.29, *p* < 0.05, FDR corrected *p* = 0.04, Cohen’s *f* = 0.35], but not on the negative subscale, general psychopathology subscale or PANSS total scores (all *p* > 0.05). In addition, there were no main effects of sex or genotype on the PANSS subscales and total scores (all *p* > 0.05). One-way MANCOVA showed that in male patients, there was a significant genotype effect on the positive subscale [*F*_(1,173)_ = 5.42, *p* < 0.05, FDR corrected *p* = 0.03, Cohen’s *f* = 0.50]. However, there was no genotype effect in female patients (*p* > 0.05). Further *post hoc* analysis (Figure 1A) indicated that male patients with Met homozygote and heterozygote had a significantly lower score than female patients with Met homozygote and heterozygote genotypes (*p* < 0.01). Male patients with Val/Val genotype had more positive symptoms than Met carriers (Val/Met vs. Met carrier: 13.3 ± 4.8 vs. 11.6 ± 4.6, *p* < 0.05), while in female patients, Met carriers exhibited more positive symptoms slightly (12.3 ± 5.4 vs. 14.7 ± 7.3, *p* = 0.07).

**TABLE 3:** Interaction of sex and *COMT* Val^158^Met (rs4680) genotypes on the clinical characteristics and cognitive performance in healthy controls and patients.

	Healthy control									Schizophrenia patients								
	Male			Female			SEX^c^F, *p*	*COMT* ^c^F, *p*	SEX × *COMT* ^c^F, *p*	Male			Female		SEX^c^F, *p*	*COMT* ^c^F, *p*		SEX × *COMT* ^c^F, *p*
										
	Val/Val (*n* = 86)	Met carrier (*n* = 82)	^a^F, *p*	Val/Val (*n* = 111)	Met carrier F(*n* = 118)	^b^F, *p*				Val/Val (*n* = 78)	Met carrier (*n* = 103)	^a^F, *p*	Val/Val (*n* = 37)	Met carrier F(*n* = 26)	^b^F, *p*			
**PANSS**	–	–	–	–	–	–	–	–	–									
Positive subscale	–	–	–	–	–	–	–	–	–	13.3 ± 4.8	11.6 ± 4.6	5.4,0.01**	12.3 ± 5.4	14.7 ± 7.3	1.8,0.18	1.4,0.24	0.4,0.54	7.3,0.01*
Negative subscale	–	–	–	–	–	–	–	–	–	22.1 ± 7	22.1 ± 7.2	0.1,0.92	21.1 ± 6.9	20 ± 8.1	1.9,0.16	2.4,0.12	0.4,0.51	0.6,0.44
General subscale	–	–	–	–	–	–	–	–	–	27.4 ± 5	25.8 ± 4.8	3.7,0.03*	27.8 ± 6.2	27.9 ± 6.2	0.2,0.81	1.8,0.18	0.9,0.34	0.9,0.36
Total	–	–	–	–	–	–	–	–	–	62.8 ± 12.8	59.5 ± 13	2.2,0.12	61.1 ± 14.1	62.6 ± 15.6	0.0,0.98	0.0,0.85	0.2,0.63	1.1,0.31
**RBANS**																		
Immediate memory	73.8 ± 16.9	76.6 ± 18.7	0.2,0.62	77 ± 16.5	75.8 ± 17	0.3,0.58	0.8,0.38	0,0.96	0.5,0.47	57.4 ± 13.1	66.8 ± 20.4	12.9,0.00**	67.3 ± 19.3	66.5 ± 20.7	0.2,0.69	4,0.05*	2,0.15	4.4,0.04*
Visuospatial/constructional	79 ± 15.7	80.5 ± 15.8	0,1	80.8 ± 16	79.4 ± 15.4	0.5,0.46	0.4,0.56	0.2,0.7	0.3,0.56	79.4 ± 20.1	85.4 ± 18.3	5.9,0.02*	87.2 ± 20.1	84.7 ± 18.6	0.5,0.47	1,0.33	0.3,0.57	3.4,0.07
Language	94.1 ± 11.1	95 ± 12.3	0.1,0.78	93.4 ± 13.8	94.3 ± 13.8	0.2,0.65	0.2,0.64	0.0,0.86	0.2,0.69	84.5 ± 13.3	88.4 ± 13	4.4,0.04*	84 ± 17.1	91 ± 13.8	2.6,0.11	0.2,0.67	6.6,0.01*	0.4,0.51
Attention	87.2 ± 20.4	88.6 ± 20.5	0,0.93	88.7 ± 19.6	87 ± 20.4	0.5,0.49	0.0,0.91	0.4,0.54	0.1,0.75	78.4 ± 14.2	82.8 ± 14.9	4.6,0.03*	84.4 ± 14.3	79.8 ± 18.1	1.6,0.21	0.4,0.53	0,0.85	4.9,0.03*
Delayed memory	86.8 ± 14.5	87.1 ± 13.7	0.2,0.66	87.6 ± 14.4	85 ± 16.5	1.7,0.19	0.0,0.92	1.7,0.19	0.3,0.62	66 ± 18.6	71.4 ± 21.3	4.3,0.03*	78.5 ± 20.2	75.5 ± 22.7	0.6,0.43	6.9,0.01*	0.1,0.78	2.9,0.09
RBANS total score	79.5 ± 14.6	81.3 ± 15.5	0.0,0.86	80.95 ± 14.8	79.8 ± 15.5	0.4,0.53	0.1,0.78	0.2,0.67	0.2,0.66	66.7 ± 12.6	73.5 ± 15.1	9.0,0.00**	75.1 ± 16.9	74.4 ± 18.2	0.2,0.61	4.3,0.04*	1.7,0.2	4.2,0.04*

^a^F: one-way MANCOVA in the male subjects.

^b^F: one-way MANCOVA in the female subjects.

^c^F: two-way MANCOVA in the health control/patients. Age and education are defined as the covariates in healthy controls; Age, age of onset, and education as the covariates in patients.

*p*: * < 0.05, ** < 0.01.

### Analysis of the interaction of sex and catechol *O* methyl transferase Val158Met (rs4680) genotype on cognitive performance in healthy controls and schizophrenia patients respectively

In patients, two-way MANCOVA with age, age of onset, and education as covariates indicated that there were significant sex effects on immediate memory [*F*_(1,235)_ = 3.98, *p* < 0.05, FDR corrected *p* = 0.06, Cohen’s *f* = 0.26], delayed memory [*F*_(1,235)_ = 6.91, *p* < 0.01, FDR corrected *p* = 0.02, Cohen’s *f* = 0.34], and total score [*F*_(1,235)_ = 4.33, *p* < 0.05, FDR corrected *p* = 0.06, Cohen’s *f* = 0.27]. Moreover, there was a significant genotype effect on language [*F*_(1,235)_ = 6.57, *p* < 0.05, FDR corrected *p* = 0.01, Cohen’s *f* = 0.33]. There was a significant sex × genotype interaction on immediate memory [*F*_(1,235)_ = 4.44, *p* < 0.05, FDR corrected *p* = 0.07, Cohen’s *f* = 0.33], attention [*F*_(1,235)_ = 4.92, *p* < 0.05, FDR corrected *p* = 0.05, Cohen’s *f* = 0.29], and total score [*F*_(1,235)_ = 4.21, *p* < 0.05, FDR corrected *p* = 0.04, Cohen’s *f* = 0.27]. This sample gave a statistical power of > 0.9.

One-way MANCOVA showed that in male patients, there was significant genotype effect on all the cognitive indexes: immediate memory [*F*_(1,175)_ = 12.9, *p* < 0.01, FDR corrected *p* = 0.002, Cohen’s *f* = 0.54], visuospatial/constructional [*F*_(1,175)_ = 5.9, *p* < 0.05, FDR corrected *p* = 0.03, Cohen’s *f* = 0.37], language [*F*_(1,175)_ = 4.4, *p* < 0.05, FDR corrected *p* = 0.05, Cohen’s *f* = 0.32], attention [*F*_(1,175)_ = 4.6, *p* < 0.01, FDR corrected *p* = 0.05, Cohen’s *f* = 0.32], delayed memory [*F*_(1,175)_ = 4.3, *p* < 0.01, FDR corrected *p* = 0.05, Cohen’s *f* = 0.31], and total score [*F*_(1,175)_ = 9.0, *p* < 0.01, FDR corrected *p* = 0.01, Cohen’s *f* = 0.45]. However, in female patients, there were no significant differences between genotypes in any of the cognitive indexes. Further *post hoc* analysis showed that male patients with Val homozygotes had the lowest score in all the cognitive indexes (Figures 1B–G).

In the healthy controls, we did not find any significant sex effects, genotype effects, or sex × genotype interactions on cognitive performance (all *p* > 0.05). Further *post hoc* analysis showed that in either male or female healthy controls, there was no significant genotype effect on any cognitive indexes (all *p* > 0.05).

### The inconsistent mediating effect of catechol *O* methyl transferase Val158Met (rs4680) polymorphism on the relationship between sex and cognitive performance in schizophrenia patients

**FIGURE 2:**
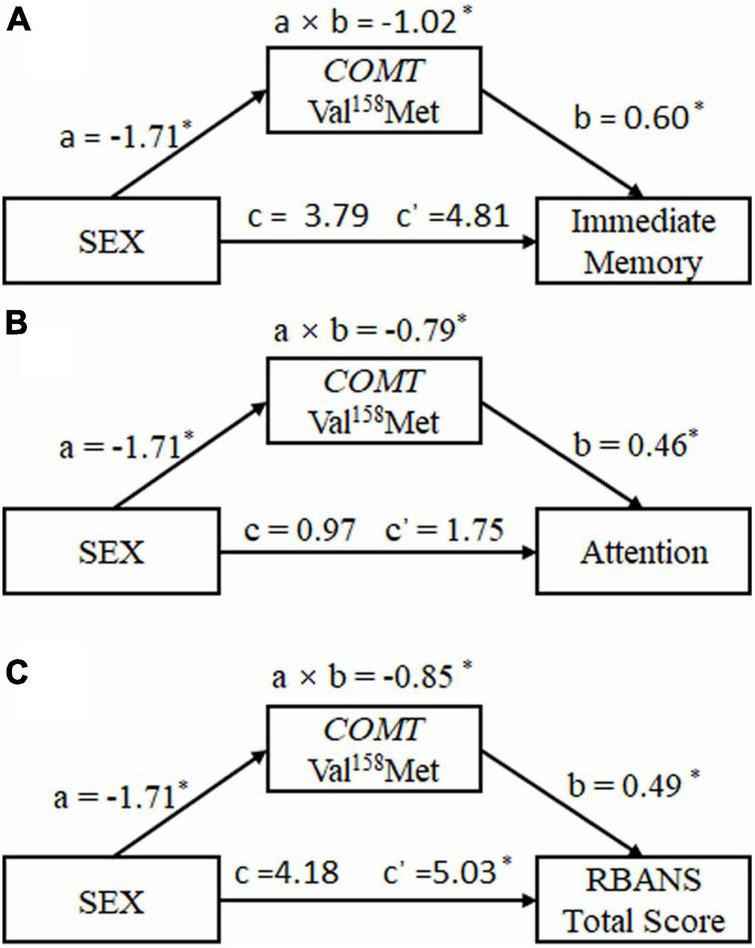
The inconsistent mediating effect of catechol *O* methyl transferase Val158Met (rs4680) polymorphism on the relationship between sex and cognitive performance in schizophrenia patients. **(A)** Immediate memory. **(B)** Attention. **(C)** RBANS total score. a × b: the indirect effect of sex on cognitive performance; c = the total effect of sex on cognitive performance; c’ = the direct effect of sex on cognitive performance; a = the effect of sex on *COMT* Val158Met (rs4680) polymorphism; b = the effect of *COMT* Val158Met (rs4680) polymorphism on cognitive performance. *p < 0.05.

Figure 2 depicts the results of the mediation models that were tested. Analysis revealed significant indirect effects of sex *via COMT* Val158Met (rs4680) on cognitive performance [immediate memory: a × b = −1.02, 95% CI (−0.59, 10.21); attention: a × b = −0.79, 95% CI (−2.74, 6.25); RBANS total score: a × b = −0.85, 95% CI (0.37, 9.69)]. Bootstrapping (resamples = 5,000) procedures were used to evaluate the significance of the indirect effects. The bootstrap confidence did not include zero, indicating significant effects. In the mediation models, c’ was opposite in sign to a × b and the total effects were non-significant, which meant that the total effect of sex on cognitive performance of schizophrenia patients was not observed because the direct effects and indirect effects cancel each out.

## Discussion

In this study, we investigated the interaction of *COMT* Val158Met (rs4680) polymorphism and sex on clinical characteristics and cognitive performance of patients with schizophrenia. The main findings of this study were as follows: (1) Sex differences in the allele frequency and genotype distribution of *COMT* Val158Met (rs4680) were found only in patients with schizophrenia. In male individuals, the patients had a significantly higher proportion of carrying the Val allele. (2) The male patients with Val/Val exhibited more positive symptoms and more severe cognitive dysfunction than male Met carriers. (3) There was an inconsistent mediating effect of *COMT* Val158Met (rs4680) polymorphism on the relationship between sex and cognitive performance in schizophrenia patients.

The results of the association between COMT Val158Met (rs4680) polymorphism and schizophrenia risk have been contradictory. Our results reveal no significant differences in allele and genotype frequencies of *COMT* Val158Met (rs4680) polymorphism between patients with schizophrenia and healthy controls. This result is in line with many other studies conducted on East Asian populations (20–22). However, the *COMT* Val158Met (rs4680) polymorphism is found to play an important role in susceptibility to schizophrenia in Caucasian and African Americans (3, 23–25). A meta-analysis report shows that there is no evidence of a significant association among the Asian populations, while the risk of disease in Caucasians with the Val allele is increased up to 10–23% (26). These pieces of evidence suggest that the association between *COMT* Val158Met (rs4680) polymorphism and schizophrenia may be race specific. However, previous studies may have ignored the impact of sex differences. In this study, we find that compared with healthy people, the distribution of Val158Met (rs4680) gene polymorphism had significant sex differences in the allele and genotype frequencies of patients.

The reported findings of sex differences in cognitive impairment in schizophrenia patients are inconsistent and, based on our findings, are likely due to the offsetting effects of sex and genetic factors (27). *COMT* Val158Met (rs4680) regulates the dopaminergic transmission in the PFC, where it accounts for more than 60% of the metabolic degradation of DA (28). COMT is abundantly found in microglial cells in the brain (29), whereas there are significant sex differences between microglia and neuroimmune signaling throughout the life span (30). At normal body temperature, the COMT activity of Val allele carriers is three times higher than that of Met allele carriers. Higher COMT activity leads to a lower DA signal, so the Val allele may cause physiological impairment of the prefrontal lobe (31). For example, Val/Val patients with schizophrenia show more severe psychiatric symptoms (32), more positive/negative symptoms (8, 21, 33), and worse performance in working memory tasks (34). Met allele load can predict improvement in cognitive performance and positive/negative symptoms after antipsychotic treatment (32, 35, 36). The current study finds that *COMT* Val158Met (rs4680) polymorphism has a sexually dimorphic effect on positive symptoms. Consistent with our study, Goghari et al. reported that male patients with Val homozygotes demonstrated greater positive symptoms than those male patients with Met carriers (33). The DA hypothesis of schizophrenia assumes that positive symptoms can be attributed to the hyperactivity of dopamine D2 receptors in the subcortical and limbic brain regions, while negative symptoms can be attributed to the hypo functionality of dopamine D1 receptors neurotransmission in PFC (37). However, little is known about the relationship between the *COMT* Val158Met (rs4680) polymorphism and DA receptors.

Dopamine (DA) has been shown to play an important role in PFC-mediated cognition (38). Evidence from *COMT* knockout mice and pharmacological investigations has confirmed the importance of COMT for dopaminergic clearance in PFC (10, 11, 31). Since the COMT efficacy of the Val alleles is three to four times higher than that of Met alleles, this difference may shape cognitive performance. In our study, male patients with homozygous Val alleles show worse cognitive performance in immediate and delayed memory, which is consistent with some previous studies. For example, Bilder et al. reported that the *COMT* Met allele was associated with better performance in processing speed and attention ability in patients with chronic schizophrenia (39). Matsuzaka et al. found that Val/Val carriers scored the lowest in working memory tasks (34). Shukla et al. found that Val homozygotes showed deficient performance on the dorsolateral-prefrontal-cortex-dependent task (40). This study finds a relationship between Val homozygotes and language. However, we find that male patients with Val/Val have an increased risk of cognitive impairment when considering the influence of sex. This sex-*COMT* interaction on cognitive function has also been verified in transgenic mice (41). The activity levels of the COMT enzyme are also influenced by sex, showing that male subjects and Val alleles are associated with higher enzyme activity and possibly lower PFC DA levels (42). Although relatively little is known about how the genetic variation of Val158Met (rs4680) affects brain structure and function, a recent study has found that male subjects with Met/Met have higher subcortical volumes (10). Male and female patients have different association patterns between the *COMT* gene and disease phenotype. The COMT effect is relatively weak among women (43). Notably, the sex-dependent effects on cognition were not observed in normal healthy people. Considering the “inverted U-shaped” relationship between DA level and cognitive function, healthy individuals may be more likely to maintain an optimal level of DA (44).

In summary, our results suggest that the Val allele of *COMT* Val158Met (rs4680) genotype is strongly associated with the positive symptoms and cognitive dysfunction of Chinese male schizophrenia patients. Undeniably, there are several limitations in the current study. (1) The influence of drugs cannot be ruled out. (2) This study only examined one candidate gene, while other potential polymorphisms may be involved in the psychopathological symptoms of schizophrenia. (3) We cannot separate the homozygous Met/Met group and the heterozygous Val/Met group from the “Met allele carriers.” Therefore, a larger sample size is needed to confirm our findings, and more studies are necessary to elucidate the mechanism in depth. In conclusion, this study comprehensively studied the sexually dimorphic effect of *COMT* Val158Met (rs4680) polymorphism in schizophrenia patients in the Chinese population. These findings could have implications for understanding the factors that may lead to different manifestations between male and female schizophrenia patients.

## Data availability statement

The original contributions presented in this study are included in the article/supplementary material, further inquiries can be directed to the corresponding author.

## Ethics statement

The studies involving human participants were reviewed and approved by the Institutional Review Board, Beijing Hui-Long-Guan Hospital. The patients/participants provided their written informed consent to participate in this study.

## Author contributions

XZ designed the study and managed study supervision. MX, DC, and YZ interviewed the participants and conducted the clinical assessment. HX analyzed the data and drafted the manuscript. HX, XZ, WW, and LW revised and completed the manuscript. All authors contributed to the article and approved the submitted version.
